# Self- and non-self-DNA on hands and sleeve cuffs

**DOI:** 10.1007/s00414-023-03124-9

**Published:** 2023-12-06

**Authors:** Léonie Henry, Martin Zieger

**Affiliations:** Institute of Forensic Medicine, Forensic Molecular Biology Department, University of Bern, Murtenstrasse 26, 3008 Bern, Switzerland

**Keywords:** DNA, Transfer, Background, Sleeve cuff, Hand, Shedder status

## Abstract

Studying DNA transfer and persistence has become increasingly important over the last decade, due to the impressive sensitivity of modern DNA detection methods in forensic genetics. To improve our understanding of background DNA that could also potentially be transferred, we analyzed the DNA composition on the outside of sleeve cuffs and sampled DNA directly from the hands of four different collaborators upon their arrival at work during 25 working days. Sampling of their hands was repeated after several hours working in our department. The shedder status of the participants, as assumed from previous internal studies, was well re-produced in the study. However, we noticed that the DNA shedding capacity could also change drastically during the day, with one participant showing a more than sixfold increase between hands sampled in the morning and hands sampled in the afternoon. As expected, poor DNA shedders carry more relative amounts of non-self-DNA on their hands than good shedders. Non-self-alleles were detected in 95% of the samples. We also observed potential effects of hand washing and the mode of transport to get to work on the DNA amount. People living with family members occasionally carried their DNA on their hands and more frequently on their sleeve cuffs. Sleeve cuffs, as being close to our hands, have a large potential to transfer DNA from one place to another, yet they have sparsely been studied as DNA transfer intermediates so far. In general, we collected consistently more DNA from the sleeve cuffs than from the hands of the participants, demonstrating their importance as potential transfer vectors. More DNA was recovered from sleeve cuffs made of synthetic fabric than from cuffs made of cotton or leather. In the afternoon, DNA from co-habitant family members could not be detected on the hands anymore and the detection of profiles from colleagues became more frequent. From two out of 100 analyzed sleeve cuffs and two out of 200 sampled hands, we established unknown major DNA profiles that would have been suitable for an entry in the national DNA database. This finding demonstrates the possibility to transfer DNA that has most likely been picked up somewhere in the public space.

**Supplementary Information:**

The online version contains supplementary material available at 10.1007/s00414-023-03124-9.

## Introduction

Advances in laboratory analysis methods have facilitated the examination of crime scene DNA traces, which mostly bear only minute amounts of DNA. The increasing sensitivity of DNA analysis has led to a growing interest in DNA transfer processes [1, 2]. A better understanding of transfer and persistence is of crucial significance in enlightening the relation between the crime committed and the DNA found at the crime scene. The amount of DNA that can be transferred depends on various factors such as the characteristics of the primary source of DNA [3], the type of surface [4], and the mode of transfer [3, 5, 6].

Several studies have been published demonstrating the possibility of transferring self- and non-self-DNA directly from hands to various items after handshaking [7, 8] or indirectly after different contacts with objects, such as knives [9, 10]. However, to our knowledge, DNA has rarely been sampled directly from hands in previous studies [11]. Yet, from a logical standpoint, we argue that DNA present on hands should be considered in the context of non-self-DNA transfer since the presence of DNA is a prerequisite for subsequent transfer. Therefore, we aimed to describe the amounts and origins of self- and non-self-DNA that can be sampled directly from hands under unsupervised conditions.

DNA cannot only be transferred by hands, as demonstrated by studies focusing, e.g., on the presence of DNA on clothing [12–14]. We noticed that, among these studies, sleeve cuffs have so far only sparsely been investigated, with the exception of one study which analyzed the inside coating of sleeve cuffs, to identify the potential wearer of a given garment [15] and a study from Szkuta et al., investigating the DNA on various parts of upper garments, including the exterior of sleeve cuffs [16]. However, the close proximity of sleeve cuffs and hands, the much lower cleaning frequency of clothing compared to hands, and the frequent contact of sleeve cuffs with various external surfaces make them strong potential DNA vectors.

The aim of this study was to assess the potential of DNA transfer, both self- and non-self, by looking directly at the amount and composition of background DNA present on hands and sleeve cuffs. We analyzed to which extend detected non-self-DNA originates from known or unknown individuals, by comparing it to DNA profiles of individuals with whom the study participants share their work or living space. We also recorded factors that may have an impact on the amount and transfer of DNA, such as hand washing, wearing of gloves, mode of transport taken to workplace, composition of sleeve cuffs, and shedder status of the participants.

## Material and methods

### Experimental setup

This study comprised of a group of four collaborators of our institute, henceforth referred to as A, B, C, and D. The participants were chosen based on their shedder status, known from previous internal studies. The group shared common work-related spaces but were neither partners nor genetically related and did not live together. All participants provided written informed consent.

Every morning, upon arrival at work, DNA from their dominant hand (right for all participants) and from the corresponding sleeve cuff of their outer-most layer of clothing (e.g., jacket or coat) were sampled. An additional DNA sample from the dominant hand was taken during the afternoon after they had spent several hours at the workplace (approximately two hours after the lunch break, resulting in a 5- to 7- h interval).

Samples were collected during 25 days, between March 6 and May 26, resulting in a total of 200 hand samples and 100 sleeve cuff samples. At this time of the year, average monthly temperatures ranged between 6.7 °C in March and 13.8 °C in May, with extreme values between − 4.2 °C and 25.6 °C. While in previous studies no significant differences were found regarding hand dominance [17–19], it was decided that using the dominant side was more appropriate since it is prone to be in contact with more objects, especially tools, and may therefore have the greater potential for case-relevant DNA transfer. Each participants’ daily routine was left unaltered. However, they were advised to avoid washing their hands upon arrival in the morning, before the sampling.

Participants’ activities preceding the morning sampling were recorded by a questionnaire. Questions included were mode of transport to the workplace, time since last handwashing before sampling, and whether gloves were worn more or less than 30 min before sampling in the afternoon. Lastly, the composition of the sampled sleeve cuff and the time period since the last washing of the piece of clothing were recorded. All DNA samples were collected by the same person equipped with lab coat, facemask, and gloves to minimize risk of contamination.

Buccal swabs from permanent co-residents of the participants were collected to obtain reference DNA profiles after written informed consent. A had two flatmates, B lived with their partner, C lived with their partner and their two children, and D lived alone. All reference samples received a non-speaking identifier, and they were processed anonymously in the lab. Samples were discarded immediately after profile generation. DNA profiles from the four participants and all other collaborators of the department were already available in the laboratory for quality assurance purposes.

### DNA sampling on hands

DNA was collected using one sterile viscose forensic swab per hand (Sarstedt, Nümbrecht, Germany). Contact with external objects or surfaces at the crime scene usually occurs in the absence of liquids, which might enhance DNA transfer. Hence, dry swabs were used for sampling to mimic a more realistic scenario. DNA collection started by swabbing the palm, followed by the fingers, starting with the fifth digit and ending with the thumb.

### DNA sampling on sleeve cuffs

DNA samples from sleeve cuffs were collected using SceneSafe Fast™ minitapes (SceneSafe, UK), as previously proposed by Hess and Haas [20]. No instructions regarding use and composition of the garments were imposed on the participants. Sleeve cuffs were sampled following three lines with seven applications each, resulting in an area of 13.3 cm × 6.6 cm, located on the back of the cuff (Suppl. Figure 1). DNA-Tapes were stored in 2-mL tubes at room temperature.

### DNA extraction and purification

All laboratory analyses were conducted according to the established standard operating procedures of our lab for swabs and DNA tapes. PrepFiler Express™ Kit (ThermoFisher Scientific, Waltham, MA, USA) was used to extract DNA from swabs. Swab heads were removed with sterile razor blades and placed in 2-mL tubes with 500 μL of PrepFiler Lysis Buffer and 5 μL of Dithiothreitol 1 M (DTT). Samples were homogenized in a Precellys® 24 Touch homogenizer (Bertin instruments, Montigny-le-Bretonneux, France) for 2 × 30 s at 5900 rpm, followed by an overnight incubation at 56 °C and 400 rpm on a thermo-shaker (Labgene Scientific SA, Châtel-Saint-Denis, Switzerland). Tube contents were transferred to tubes including spin baskets for centrifugation to separate the cell lysate from the swab heads. Finally, DNA purification was performed using an AutoMate Express™ Nucleic Acid DNA Extraction System (Thermo Fisher Scientific, Waltham, MA, USA), with an elution volume of 50 μL. Extracted DNA was stored at 4 °C before subsequent analyses.

Stoop et al. [21] demonstrated that DNA recovery from the SceneSafe Fast™ Tapes by extraction with phenol–chloroform is more efficient than using magnetic bead-based extraction protocols. Therefore, tapes were cut in 10–12 pieces with sterile razor blades and incubated in a mix of 450 μL of stain extraction buffer (SEB; pH 8.0; 10 mM Tris, 10 mM EDTA, 100 mM NaCl), 50 μL of sodium dodecyl sulfate (SDS; 10%), 10 μL of proteinase K (20 mg/mL), and 10 μL of dithiothreitol (DTT; 1 M). Samples were incubated at 56 °C overnight on a thermo-shaker at 400 rpm (Labgene Scientific SA, Châtel-Saint-Denis, Switzerland). Then, 5 μL of proteinase K (20 mg/mL) was added, followed by an additional incubation of at least 2 h. The pieces of tapes were removed from the cell lysate and transferred to tubes including spin baskets for centrifugation. After adding 800 μL of phenol:chloroform:3-methylbutane-1-ol 25:24:1 (Sigma-Aldrich, US) to the lysate, the aqueous phase was transferred with distilled water in a Vivacon® 2 ETO column (Vivaproducts, Inc., US) for cleaning, as described previously [21]. Extracted DNA was stored at 4 °C before subsequent analyses.

### DNA quantification, amplification, and data analysis

DNA quantification was performed on a 7500 Real-Time PCR System (Thermo Fisher, Waltham, MA, USA), with the Quantifiler™ HP Kit according to manufacturer’s instructions. Results from qPCR were analyzed using the HID Real-Time PCR Analysis Software version 1.2.

Multiplex PCR was performed in reaction volumes of 25 μL using the AmpFLSTR™ NGM Select™ PCR Amplification Kit (ThermoFisher, Waltham, MA, USA), on a T3000 Biometra Thermocycler (Analytik Jena, Jena, Germany). Samples were amplified with 30 cycles by default, but 32 cycles were applied for concentrations below 0.02 ng/μL following standard internal procedures for routine casework. The optimal input amount of DNA required for multiplex PCR is 0.5 ng; therefore, a maximum sample volume of 10 μL was used for samples with concentrations below 0.05 ng/μL. For cost reasons, a single amplification was performed for all samples. For real casework samples, the Swiss law prescribes a second amplification [22].

PCR products were separated on a 3500 × L Genetic analyzer (ThermoFisher Scientific, Waltham, MA, USA), and the resulting profiles were analyzed using the software GeneMapper™ ID-X version 1.6 (ThermoFisher Scientific, Waltham, MA, USA). Profiles with at least 10 detected alleles were analyzed further. The maximum allele count method was used to determine the number of contributors. Profiles were deconvoluted and compared to the profiles from all the participants, the participants’ co-residents and from all collaborators of the department through the database search function in STRmix™ (version 2.9.1), using a Swiss reference population [23] and a F_ST_ value of 0. A real contribution to the profile (i.e., an inclusion) was assumed for all individuals with likelihood ratios (LRs) over 1000. During the deconvolution, STRmix™ estimates the respective mixture proportions of the different contributors. Using the “LR from previous” function, we compared all deconvoluted trace profiles to the profile from the respective participant from which they were sampled, to estimate the percentage of self-DNA and non-self-DNA in the samples. If STRmix proposed for a three-person mixture, e.g., mixture proportions of 65%, 25%, and 10%, and the reference profile of the participant has been attributed by the software to the 65% proportion; then, self-DNA for this sample was counted as 65% and non-self-DNA as 35%.

In forensic casework, evaluating matching profiles through probabilistic genotyping is only possible if the respective reference person, or more specifically the suspect, is known. However, this is often or even mostly not the case. Therefore, the purpose of most DNA profiles established from crime scene samples is to run a candidate search in a national DNA database. Regulated by law, Switzerland has defined the 16 STR loci, amplified by the AmpFLSTR™ NGM Select™ Kit, as database loci [22]. In the present study, we focused on major components originating from a single person, for which the criteria for an entry on the Swiss CODIS (Combined DNA Index System) database is a minimum of six typed loci. To consider loci of a single major profile as reliably typed, we used the deconvolution function of the software STRmix™ to call major components without a potential investigator bias. The probability threshold to assign an individual genotype at a given locus was kept at 99%. From internal experience, we know that a 99% probability threshold to call genotypes in the STRmix deconvolution tends to be more stringent than a manual assessment by DNA experts. Therefore, we would expect slightly more major components assigned manually by an expert in real casework, and more reliable allele calling due to PCR replicates. In addition, we did not assess our data for CODIS suitable two-person mixtures, which could also enter the Swiss national DNA database and which in real-case scenarios, could also provide us with additional investigative leads.

Statistical analyses were performed with R version 4.3.0, in Rstudio [24], using the packages “ggplot2,” “tidyverse,” “readxl,” “gdata,” “hrbrthemes,” “viridis,” and “ggpubr.” DNA amounts were log_10_-transformed and Shapiro–Wilk tests were performed to check for normal data distribution. Depending on the number of variables, *t*-tests or analyses of variance (ANOVA) were performed to check for significant differences in the detected DNA amounts. Samples that yielded an undetected amount of DNA were assigned a value of 0.0025 ng (− 2.6 on a log_10_ scale), corresponding to half the quantification limit of the Quantifiler™ HP Quantification Kit.

## Results

### DNA amounts

**Fig. 1:**
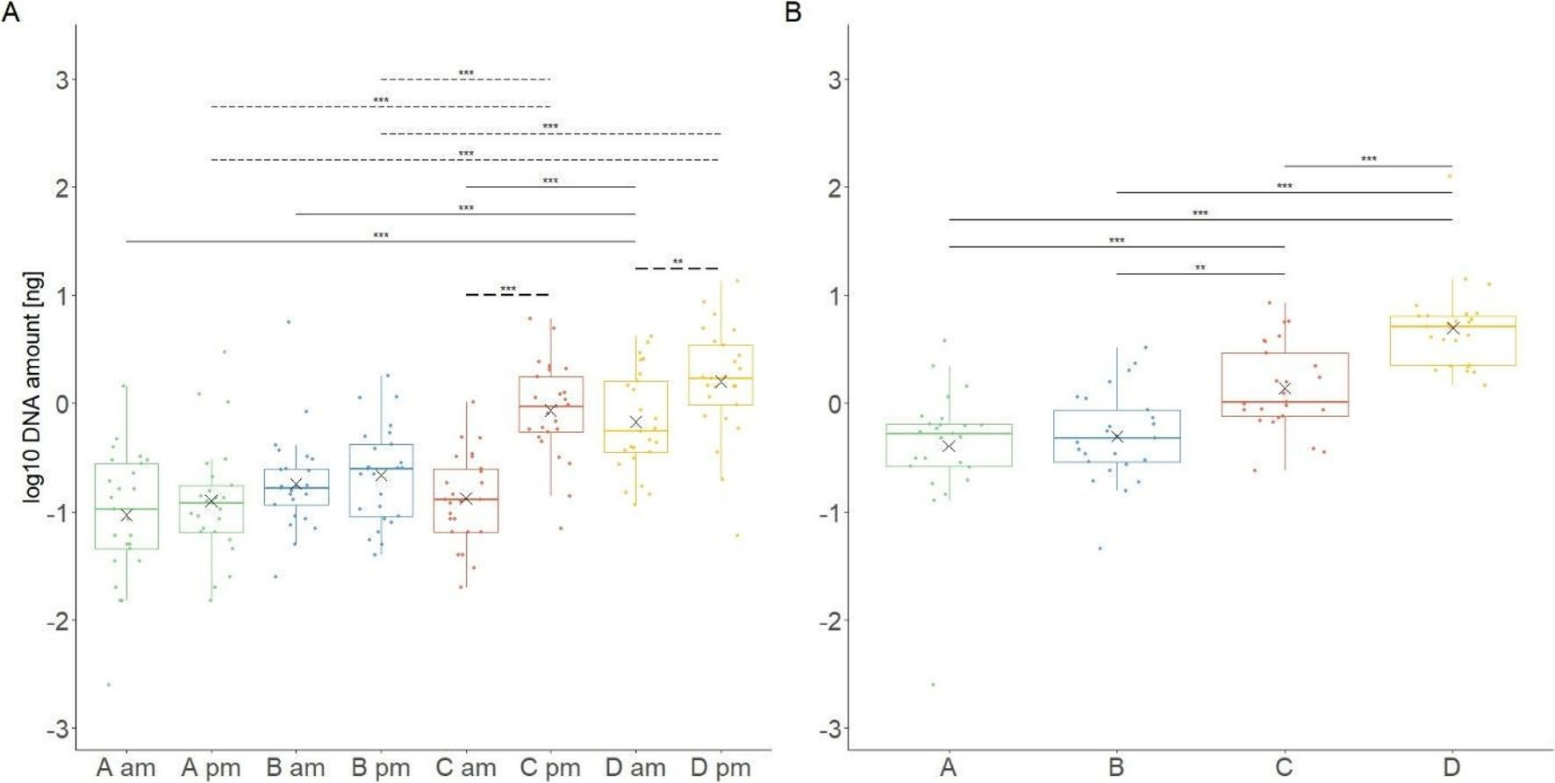
Boxplots of total log10 DNA amounts detected on **A** hands in the morning (am) and in the afternoon (pm) and **B** on sleeve cuffs of participants A, B, C, and D. 200 samples were analyzed for the hands (50 per participant) and 100 samples for sleeve cuffs (25 per participant). Black crosses represent mean values. Significant differences (***p < 0.001 and **p < 0.01) between participants are shown using thin black lines for the samples in the morning and thin black dotted lines for the samples in the afternoon. The thick black dotted lines (**A**) represent significant differences between morning and afternoon samples for the same participant

Total amounts of DNA detected on hands and sleeve cuffs of the participants are shown in Fig. 1. On two samples from A, one from the hand in the morning and one from the sleeve cuff, no DNA could be detected by qPCR. On hands, the total amounts of DNA varied significantly between morning and afternoon samples for C (*p*-value = 1.91e-08) and D (*p*-value = 9.72e-03), with higher amounts detected in the afternoon. Total amounts of DNA detected from D were always significantly higher compared to all other participants (except for the samples from C in the afternoon), whereas no significant difference in DNA amounts recovered from the hands and the sleeve cuffs between A and B could be detected. We consistently collected a higher total amount of DNA on sleeve cuffs than on hands, irrespective of the sampling time during the day (all *p*-values < 0.05), except for the samples on the hand of C in the afternoon (*p*-value = 0.091).

Whether a participant last washed their hands more or less than one hour prior to sampling had no significant impact on the detected total DNA amounts, except for C (*p*-value = 8.89e-08), with more DNA detected when the hands were washed longer ago. To assess the impact of hand washing, we pooled the data from morning and afternoon samples, to obtain greater data coverage for all participants. A summary of the total number of samples for each investigated category is shown in suppl. table 1.

For C, the total amount of DNA detected on the hand was increased on days they commuted by public transport (bus and/or train) (*n* = 8), compared to traveling by bike (*n* = 16; *p*-value = 0.0193). The same trend was found for DNA detected on their sleeve cuffs, but the results were not significant (*p*-value = 0.100). The one value for the transport to work in a private car was removed from this analysis. The other participants always commuted by public transport.

B was the only one who performed lab work on every sampling day. Whether the participant removed the disposable gloves more than 30 min (*n* = 13) or less than 30 min (*n* = 12) before the sampling in the afternoon, did not influence the total amount of DNA recovered (*p*-value = 0.650).

The composition of sleeve cuffs was grouped into “natural” (cotton, leather), “synthetic” (polyester, nylon, polyamide), and “mixed” (a mixture of natural and synthetic fibers). The amount of DNA on synthetic (*n* = 67) sleeve cuffs was significantly higher in comparison to natural fabric (*n* = 13; *p* = 0.015). However, no significant differences were detected between either “natural” or “synthetic” and sleeve cuffs classified as “mixed” (*n* = 20). All sampled pieces of clothing were washed for the last time several months ago; thus, no statistical analysis was performed to consider the time elapsed since the last washing.

### Proportions of self- and non-self-DNA

**Fig. 2:**
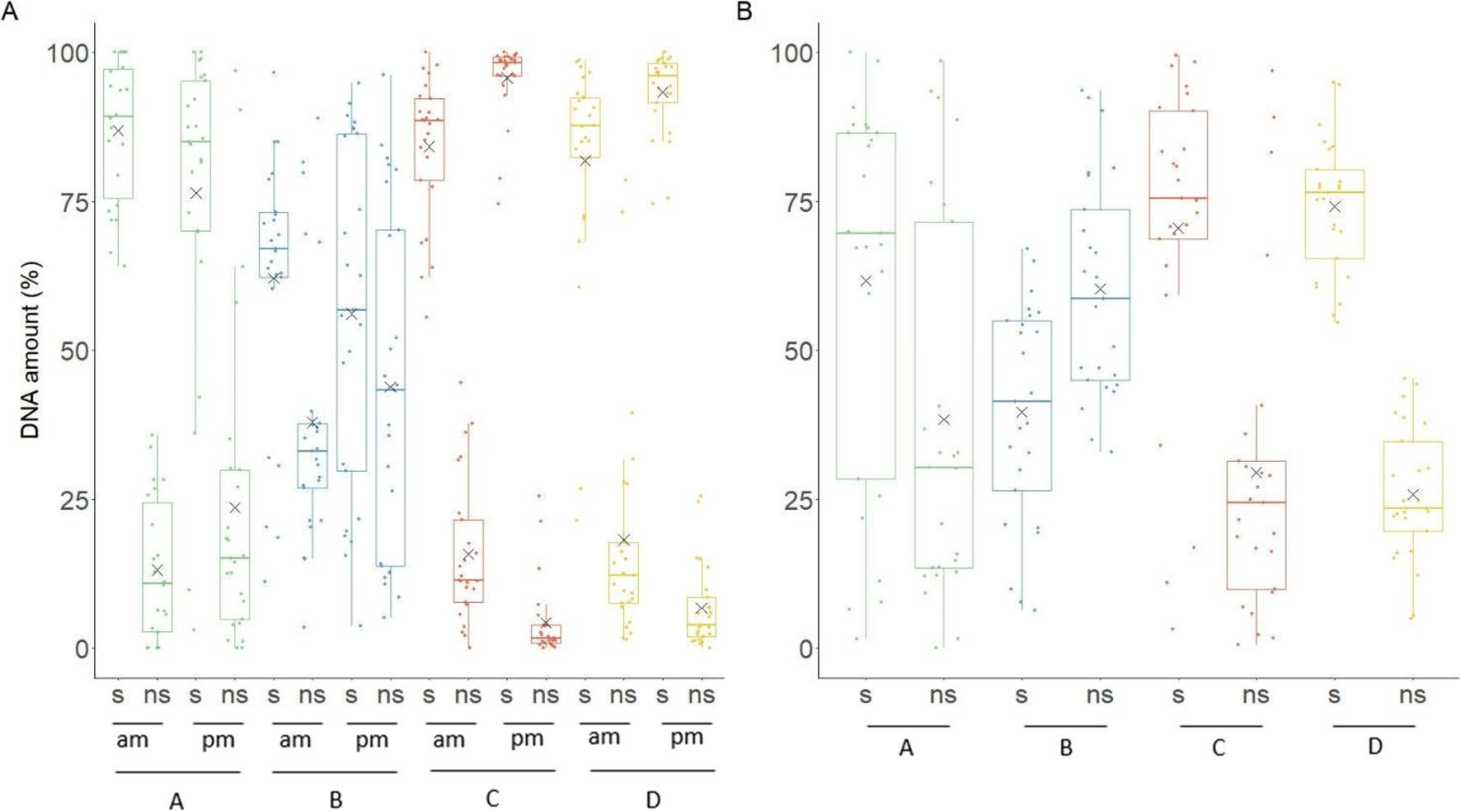
Boxplots of the percentage of self (s) and non-self (ns) DNA detected **A** on hands in the morning (am) and in the afternoon (pm) and **B** on sleeve cuffs of participants A, B, C, and D. 97 samples were analyzed for the hands in the morning (22 for participant A and 25 for participants B, C, D), 100 samples for the afternoon (25 per participant), and 100 samples for sleeve cuffs (25 per participant). Black crosses represent mean values

Percentage of self and non-self-DNA detected on hands and sleeve cuffs are shown in Fig. 2. Three of the 300 profiles, all belonging to the morning samples of A, were not interpreted because less than 10 alleles were detected. On average, the percentage of self-DNA was higher for the samples from hands in the morning than from the hands sampled in the afternoon for A and B. The opposite applied for C and D. Consistently more non-self-DNA was detected on sleeve cuffs. On average, the percentage of self-DNA detected was always higher than the percentage of non-self-DNA (except for the samples of the sleeve cuffs of B), with differences between percentages of self- and non-self-DNA much higher for C and D than A and B. The occurrence of higher percentages of non-self-DNA than self-DNA detected on hands in the morning varied between participants: 0% of samples for A and C, 20% for B, and 8% for D. In the afternoon: 16% of samples for A, 40% for B, 0% for C, and D. For sleeve cuffs: 28% of samples for A, 60% for B, 16% for C, and 0% for D. In 10.5% of all hand samples and 26% of all sleeve cuff samples, there was more non-self than self-DNA. Details on the amount of self- and non-self-DNA for every sample are listed in the Supplementary Material.

### Contributions from known individuals

The complexity of DNA profiles ranged from single source to four person mixtures (Table 1). The number of contributors tends to be higher in the samples from the sleeve cuffs. However, this effect was not observed for B. In addition, unlike the other participants, B often had DNA of four contributors on their hand and sleeve samples and never DNA of a single contributor.

**Table 1:**
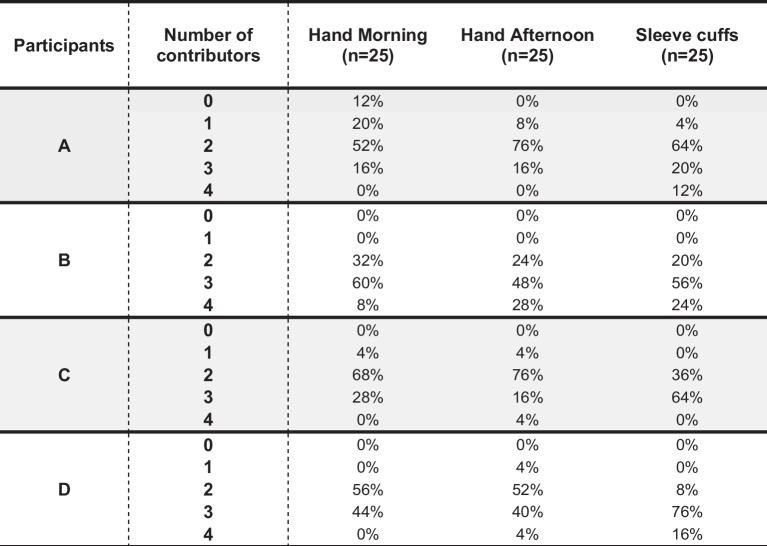
Complexity of DNA profiles as measured by the minimal number of contributors on the hands (morning and afternoon) and sleeve cuffs. The three profiles from the hands of A with less than 10 alleles are represented in the table with a number of contributors of zero

**Fig. 3:**
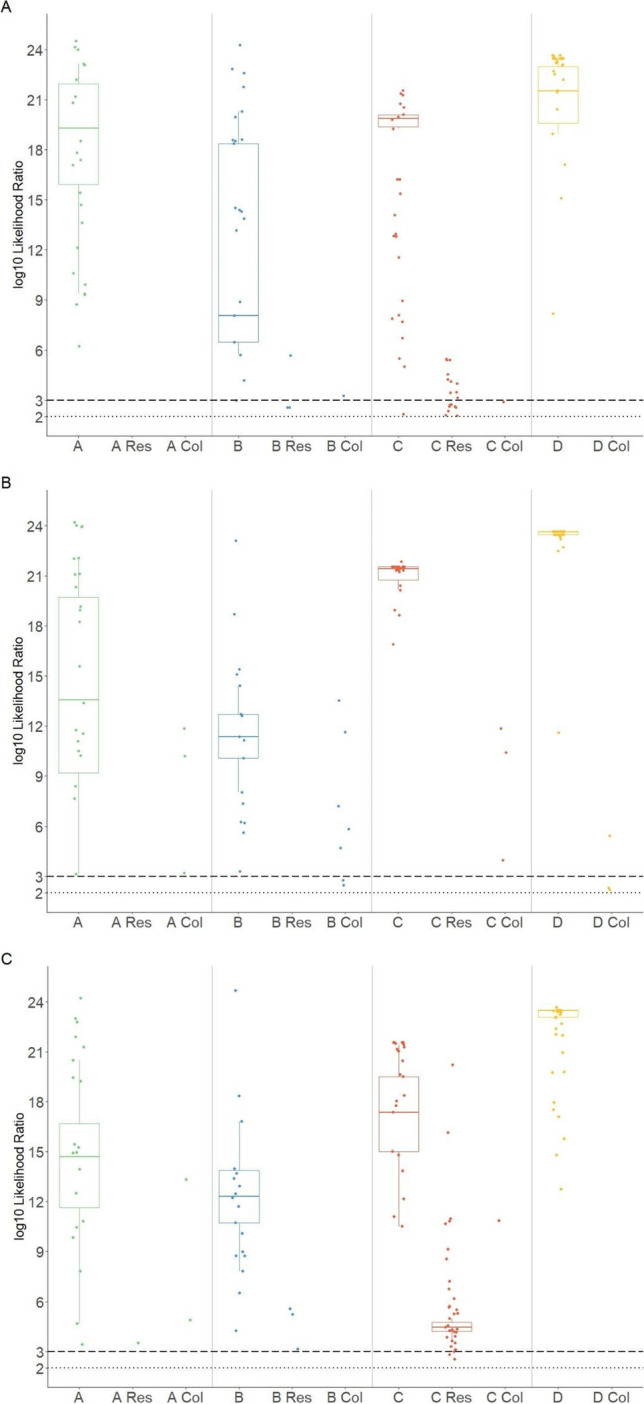
Log10 likelihood ratio (LR) values obtained from hand samples **A** in the morning, **B** in the afternoon, and **C** sleeve cuffs for participants A, B, C, and D. Log10 LR values of co-residents and collaborators of the corresponding participants are annotated wit “Res” and “Col,” respectively. The thick black dotted line is at LR value of 1000 (log10 LR = 3), corresponding to the selected inclusion threshold. Values below 100 (log10 LR = 2; thin black dotted line) are not shown

LRs for the contribution of participants, their respective co-residents, and the collaborators to the DNA profiles are shown in Fig. 3. A threshold of log_10_ LR ≥ 3 was set to assume a real contribution to the profile. The respective participants were included as profile contributors for 76 to 100% of samples taken from their hands or sleeve cuffs, with D being the only one to be included as a contributor to all their samples. At least one other individual, co-resident or collaborator, was included as a contributor in 13% of all samples.

In the morning, DNA from the co-resident of B was found one time on their hand, and 10 times for the co-residents of C (Fig. 3A). However, all co-residents included as DNA contributors in the morning were no longer present in the samples taken in the afternoon (Fig. 3B). In addition, DNA of the co-residents of participants was more often found on sleeve cuffs than on hands, especially for participant C, with 29 known non-self-contributors detected on the 25 sleeve cuffs, compared to 10 on 50 samples from their hand. However, it is possible, that some of the inclusions of C’s children are false positives, caused by increased allele sharing due to genetic relatedness. DNA of the co-residents of A could only be detected in a single sleeve cuff sample.

The DNA of collaborators was never detected on the hands of A, C, and D in the morning. This occurred only once for B. DNA of the collaborators was detected more often in the afternoon: three for A, five for B, three for C, and one for D. We detected the DNA profile of a collaborator on the sleeve cuff samples twice for A and once for C.

### Major contributor

We determined CODIS suitable major profiles using the deconvolution function of STRmix. A locus was considered database suitable, if the genotype assignment by STRmix for the first contributor exceeded the 99% probability threshold for both alleles. Controlling the called genotypes manually, in seven samples, we could clearly detect the profiles of participants as major contributors when the software falsely assigned homozygous instead of heterozygous alleles due to dropouts. The seven profiles were consequently counted as major profiles from participants and not as unknown. Major contributor profiles were found in 43.7% of all samples. The proportions of major contributor profiles varied among participants with 23% of samples for A, 13% for B, 60% for C, and 80% for D. Unknown DNA as major contribution made up 1.67% of all samples (Table 2). One of the two unknown major profiles detected on the hand of B in the afternoon could be attributed to a forensic buccal swab sample processed by B on the same day. Co-residents and collaborators were never detected as major contributors. With the exception of A, major profiles of participants could more frequently be established from the hand samples collected in the afternoon than in the morning or from sleeve cuff samples.

**Table 2:**
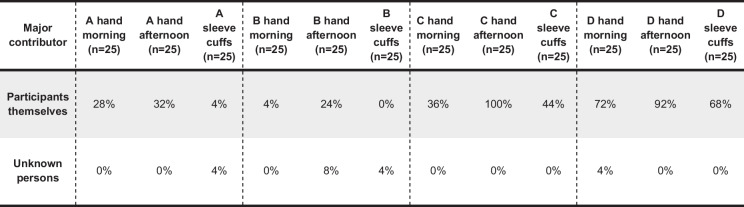
Percentages of profiles with a CODIS suitable major contributor (participants or unknown DNA), on the hands (morning and afternoon) and sleeve cuffs. No collaborators or co-residents were detected as database suitable major contributors

## Discussion

Total DNA amounts on the hands were higher in the afternoon than in the morning and varied significantly for C and D, both working mainly in an office space. This might be due to participants picking up more DNA during afternoon activities, as has previously been described [16]. According to Tan et al. [25], touching, e.g., a phone or parts of the body increases the total amount of DNA on the hands. The similarity in DNA amount between morning and afternoon samples for B might be explained by them wearing gloves during laboratory work, which may have prevented the accumulation of DNA. However, A did not accumulate significant amounts of DNA on their hand, even in the absence of gloves. These observations rather correlate with the previously established shedder status of each participant, with two good shedders, C and D, accumulating DNA over time and two poor shedders, A and B, not.

The finding for B, that wearing gloves did not significantly influence the total amount of DNA recovered, is not in line with a study which showed that the total amount of DNA deposited is significantly lower when gloves were worn within four hours prior to deposition [19]. However, this discordance may be due to the difference in chosen time periods (30 min vs. up to 4 h) and our findings being based on a single person, classified to be a relatively poor DNA shedder.

Hand washing significantly decreased the amount of DNA for C, consistent with what has been described previously [5]. For the three other participants, no significant difference was found due to handwashing, irrespective of the time period since the last wash, which in turn is in accordance with the results published by Goray et al. [19]. We therefore assume that the effect of handwashing may depend on the individual, on how the DNA-is “loaded” on their hands, and possibly on their hand washing routine and products used. Further research accounting for such individual variables could help decipher the effect of hand washing on DNA load and transfer.

The presence of various confounding factors renders it challenging to quantify which activity bears the strongest impact on total DNA amounts. Differences in DNA amounts might be due to mode of transport chosen or the individual activities during office and/or laboratory work. Taking public transport for instance leads to an elevated contact with exposed objects and surfaces in the public space and therefore may increase the DNA amounts on hands and sleeve cuffs. On the other hand, C commonly wore cotton gloves during their travels by bike, which could as well account for the highly decreased amounts of DNA on their hand in the morning compared to the afternoon. The wearing of cotton gloves and commuting by bike were often correlated, which lead to a large overlap in our dataset and therefore calls for caution in our interpretations. Similar caution has to be used when interpreting the significant differences associated with hand washing for C. Out of 25 events when hand washing occurred less than 1 h ago, 21 were in the morning, this overlap rendering it difficult to tell whether the observed difference is indeed due to hand washing or to the alternative activities carried out by C between morning and afternoon.

In average, we detected more DNA on sleeve cuffs than on hands. Given the proximity of sleeve cuffs and hands, this DNA could theoretically be readily transferred to various surfaces e.g., by manipulating tools, door handles, and so on. We recovered significantly less DNA from sleeve cuffs made of natural fabric, in comparison to synthetic materials, which was also observed by Szkuta et al. [16]. Alketbi showed that the total amount of DNA collected was significantly higher on pieces of clothing composed of 65% polyester and 35% cotton than on 100% woven cotton [13]. Our results also fit our expectation that it is more likely to recover DNA from a fabric composed of smoother and more regular synthetic fibers.

Percentages of self and non-self-DNA detected in our samples confirm that self-DNA is more likely to be detected in high proportions in comparison to non-self-DNA, as expected. Nevertheless, non-self-DNA that could potentially be transferred should not be neglected since we detected it in 95% of our samples. We consistently detected more non-self-DNA on sleeve cuffs than on hands, which is to be expected as clothing such as jackets and coats are cleaned less frequently than hands.

The co-resident status and relationship with the individual participants had a great impact on the detection of non-self-DNA originating from known individuals. The differences in co-resident detection between A on one side and B and C on the other are best explained by the nature of physical interactions, which are commonly more frequent in partnerships and in parent–child relationships than between flat mates. Thus, our results support the assumption that the DNA of co-residents is often present on day-to-day clothing [16], but that the amount and frequency largely depends on the nature of the relationship.

In the afternoon samples, DNA of B’s co-worker with whom they share the same laboratory space was found six times on B’s hand. DNA profiles from collaborators were less frequently found on the hands of the other participants what can be explained by the fact that they are mainly working on a stationary office desk.

On one of A’s sleeve cuff samples the DNA of their collaborator sharing the same office space and coat rack was detected. The sampled sleeve cuff was from a jacket that had already been worn by A the two previous days. In two other cases, DNA of collaborators was also detected on the sleeve cuffs without the clothing being worn in the days prior to sampling. This suggests that the DNA of the collaborators either remained on the sleeve cuffs for several days or that it had been picked up just moments before sampling, e.g., while opening the door to access the department.

DNA originating neither from the co-residents nor the collaborators was detected as CODIS suitable major components at five instances. One of those cases was a contamination from a buccal swab sample on the hand of B. Thus, even if unknown major profiles were observed in only 1.3% of our samples, we should not disregard the possibility to detect transferred DNA on our hands or clothes from outside sources, such as a holding bar in public transport. The majority of unknown major profiles were detected in samples taken from poor shedders, consistent with what would be expected and what has been shown previously [19].

At first glance, it seems discordant that we observe database suitable non-self-major profiles in only 1.7% of all profiles, but more non-self-DNA than self-DNA in 15.8% of all samples. However, this can be explained by the large proportion of samples with a high number of contributors and a low DNA amount, for which no database suitable major profiles can be distinguished. The 2% samples from sleeve cuffs showing a non-self-major profile correspond very well with the study of Szkuta et al. who observed a non-self-major profile in one out of 48 external sleeve cuff samples, equaling also a frequency of 2% [16].

Total DNA amounts and the presence of major contributors varied significantly among participants, supporting the idea that distinct people leave behind different amounts of DNA. The difference in proportions of self and non-self-DNA on hands was much higher for C and D and lower for A and B. These findings are in alignment with the previously assumed shedder statuses of each participant and are in agreement with the results of another study, demonstrating that a larger proportion of non-self-DNA can be recovered from hand prints deposited by poor shedders [19]. Multiple factors (e.g., gender, age, lifestyle) have been described to influence the shedder status [25–29]. Still, to which extend each of those factors can determine the amount of DNA an individual sheds remains still hardly known [26].

The mean total amount of DNA detected from the samples of C’s hand in the afternoon was 6.6 times higher than in the morning. This demonstrates that the activities carried out by an individual may have a greater impact on the total amount of DNA than their shedder status, or, turned the other way round, that the shedder status of an individual is not static and may change over a short time. Relative to the other participants, C could be classified as a bad shedder in the morning and as a good shedder in the afternoon. Repeated contact with other parts of the body, e.g., head and face, may lead to an increased DNA load on hands, which may apply to the case of C, as they reported to frequently touch their scalp during the day.

To expand our knowledge on the increase and decrease of DNA detectability over time, it would be of interest to look at the DNA on other pieces of clothing, such as shirts and pullovers, worn throughout working days. It has already been demonstrated that after wearing a piece of clothing for a day, the DNA quantity increased [29]. Shirts and pullovers are worn over longer periods but they are also washed more regularly than the jackets and coats sampled in our study. Therefore, and following our findings on hand samples, we would expect to see an accumulation of collaborator DNA during the day, but to varying degrees.

## Conclusion

The results of this study provide a better understanding of the DNA present on hands and sleeve cuffs and therefore also of the potential to transfer self- and non-self-DNA. We detected more non-self than self-DNA in 15.8% of our samples, and we detected database suitable, major profiles from unknown individuals on two out of 100 analyzed sleeve cuffs and on two out of 200 sampled hands. This highlights the potential to also transfer non-self-DNA, that has most likely been picked up somewhere in a public space, to a potential crime scene. In addition, even though not detected as a major component in this study, we should always be aware of the possibility to transfer the DNA of the people around us, especially if they get physically close to us, like in a family.

### Supplementary Information

Below is the link to the electronic supplementary material.

Supplementary file1 (XLSX 42 KB)

Supplementary file2 (DOCX 186 KB)

## Data Availability

All available data is included in the Supplementary Information.
