# Supplementary material for: Self- and non-self-DNA on hands and sleeve cuffs
Source: Int J Legal Med. 2023 Dec 6;138(3):757–66. doi: 10.1007/s00414-023-03124-9 (PMC11004043; doi:10.1007/s00414-023-03124-9)
Supplement: Supplementary file 2 — Supplementary file2 (DOCX 186 KB) [file 414_2023_3124_MOESM2_ESM.docx]

Supplementary material to:

Henry and Zieger, *Self and non-self DNA on hands and sleeve cuffs*


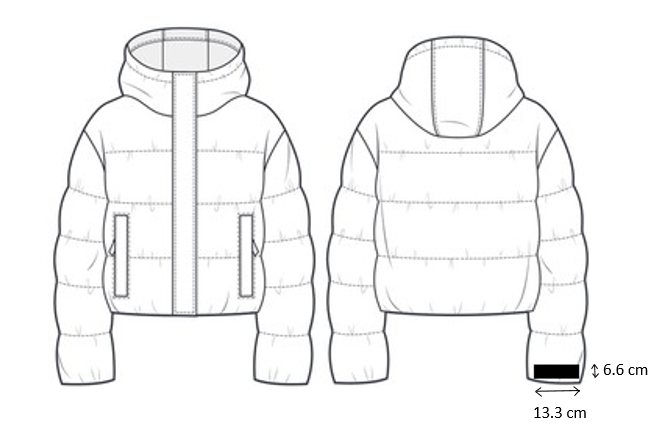


**Suppl. Fig. 1.** Sampling area on the back side of the right sleeve cuff of the upper garment of the participants


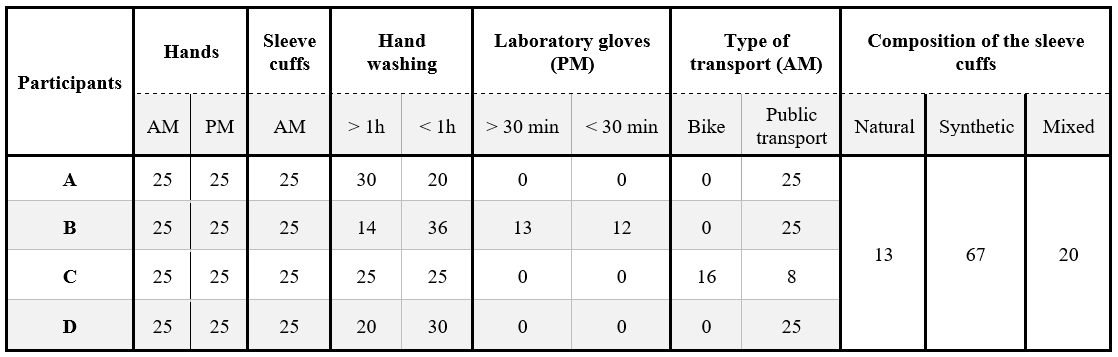


**Suppl. Table 1.** Summary of the total number of samples for each participant and recorded factors. Sleeve cuffs of different compositions were grouped over all participants.
